# BacQuant: A Scalable Automated Image Processing Pipeline for Quantifying Biofilm Aggregates

**DOI:** 10.17912/micropub.biology.002045

**Published:** 2026-07-02

**Authors:** Tevin Flom, Umur A. Ciftci, Caitlin J. Light, Whitni K. Redman

**Affiliations:** 1 First-year Research Immersion Program, Binghamton University; 2 Department of Biological Sciences, Binghamton University

## Abstract

Traditional microbiology methods rely on viable plate counting to quantify bacterial populations but often underestimate biofilm cell density because matrix-encased aggregates can produce a single colony despite containing many cells. Here, we present BacQuant, a computer vision pipeline developed in Python and OpenCV to quantify biofilm aggregates from brightfield microscopy images. Using image thresholding, segmentation, and contour detection, BacQuant distinguishes individual cells from aggregates and estimates total cell burden more comprehensively than viable plate counting alone. Automated counts closely matched manual microscopy counts while producing higher estimated densities, highlighting BacQuant as a scalable, inexpensive complimentary method for biofilm quantification.

**Figure 1. Work Flow and Representative Results of BacQuant:** **(A) Biofilm growth, sample preparation, and analysis.** 48-hour PAO1 biofilms were collected, rinsed to removed unattached cells, and sonicated to liberate biofilm-encased bacteria. The resulting suspension were mounted on slides, stained with safranin, and imaged by brightfield microscopy. Images were analyzed using the BacQuant pipeline to distinguish free cells from aggregates and determine cell counts. In parallel, cell viability was assessed by serial dilution and plating to obtain CFU/mL measurements. **(B) Representative images of stained cell populations.** Cells were stained with a safranin-based counterstain for one minute in order to show both free cell and aggregate morphologies. Examples of free cells (A) and aggregates (B) are shown by the labeled arrows. These images were typical of the aforementioned experimental design. Representative of n= 32 images. Scale bars represent 10 µm. **(C,D) Validation of the Pipeline Against Ground-Truth Labels.** Estimation plots of free cells **(C)** and aggregates **(D)** are shown. Ground truth counts were obtained by manually counting the number of free cells and aggregates in each image. The ground truth counts across 10 representative images from the total experimental set of 300 images were measured, and the pipeline was run once on each sample image. The green circles represent ground truth counts, and orange circles represent BacQuant measurements. **(E,F)** **Comparison of BacQuant Against Viable Plate Cell Counts.** Viable plate counts were determined via serial dilution and drop-plating of the post-sonicated sample. Representative images of drop-plates and corresponding microscopy images **(E)** , along with the CFU/mL calculated from BacQuant (left column) and viable plate count (right column). Average cells/mL counts from BacQuant (orange bar) were compared against CFU/mL counts from viable plate counts (blue) in **(F** ). The estimation plots above were generated from paired t-tests.; ****, p<0.0001, n=10. Statistical significance was determined with an unpaired t-test, and error bars represent standard deviation. ****, p<0.0001, n=18-22 biofilm samples with 10 images per sample.

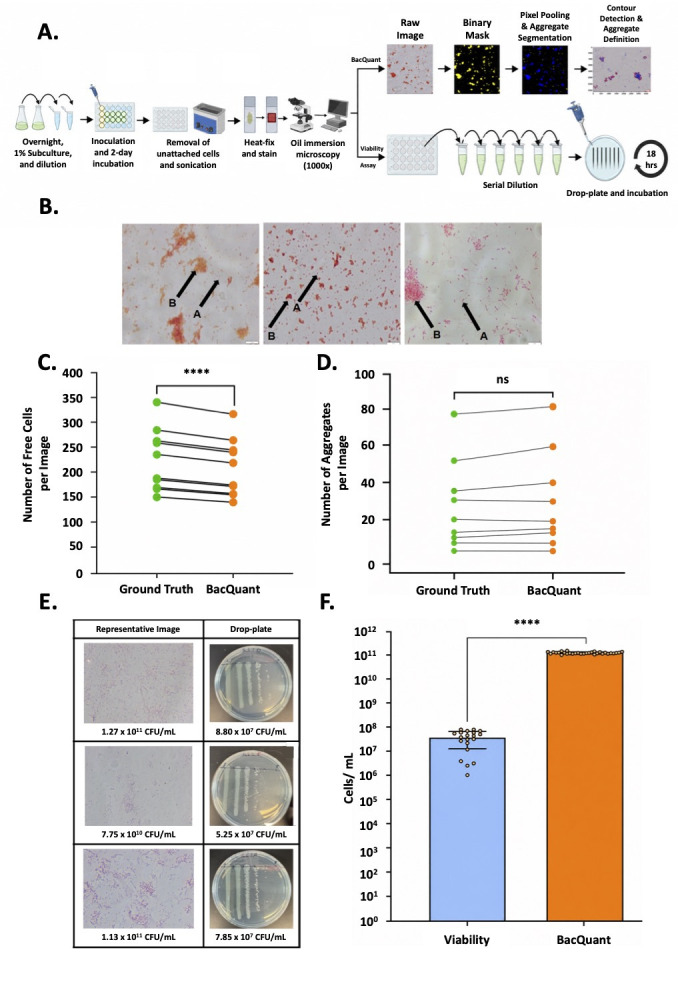



## Description

Biofilms are communities of bacteria encased in a matrix composed of extracellular polymeric substances (EPS) including lipids, proteins, extracellular DNA, and polysaccharides (Flemming et al., 2025). These communities are ubiquitous across medical, industrial, and environmental systems and are responsible for an estimated $4 trillion in global economic damage annually (Camara et al., 2022). Clinically, biofilms contribute to approximately 80% of human infections, where the EPS confers protection from antibiotics and host immune responses (Fedorowski, Moller, & Melander, 2013). Both Gram-positive species, such as *Staphylococcus aureus,* and Gram-negative species, such as *Pseudomonas aeruginosa,* exhibit biofilm-associated tolerance and persistence that complicate treatment (Schaber et al., 2007; Usui, Yoshii, Thiriet-Rupert, Ghigo, & Beloin, 2023).

A major challenge in biofilm research is accurate quantification of bacterial populations following experimental treatment. Conventional colony-forming unit (CFU) or viable plate counting (VPC) methods remain widely used because they specifically measure culturable, viable bacteria. However, in biofilm samples, VPC may not fully reflect the total number of cells present because aggregates containing multiple cells can produce a single colony if they are not completely freed from the matrix before plating (Beal et al., 2020; Martini, Boddu, Nemenman, & Vega, 2024). Mechanical disruption methods, such as homogenization or sonication, are commonly used to break up biofilms prior to plating. While optimized detachment and sonication protocols can substantially improve biofilm separation, incomplete dissociation of EPS-bound aggregates may still occur depending on the organism, biofilm structure, treatments, and disruption protocol used (Buckingham-Meyer et al., 2022; Korshoj & Kielian, 2024).

Recent advances in computer vision and microscopy offer alternative strategies for microbial quantification. Image-based methods can distinguish individual cells from aggregates using segmentation and contour detection, providing structural information that is lost in culture-based approaches (Holicheva et al., 2025; J. Wang et al., 2022). However, existing tools such as BiofilmQ and confocal-based pipelines are computationally intensive, require specialized hardware, or are not designed for post-disruption enumeration (Hartmann et al., 2021; Mountcastle et al., 2021).

Here, we present BacQuant, a computationally inexpensive image processing pipeline to improve quantification of biofilm cells following sonication using brightfield microscopy. Using *P. aeruginosa* as a model organism, BacQuant differentiates free cells from aggregates and estimates aggregate cell numbers to improve quantification relative to VPC methods **( Figure 1A ).** It is important to note that BacQuant does not intend to count viable cells. Rather, it estimates the total cell count in the biofilm sample including both live and dead cells. It does not estimate live cells like traditional VPC methods do.

Sonication of *P. aeruginosa* biofilms consistently yields two morphologies – free individual cells and EPS-encased aggregates ( **Figure 1B** ). Operationally, BacQuant defines these categories by segmented object size rather than by visual interpretation alone. Free cells displayed relatively uniform size and shape, whereas aggregates varied widely in morphology, often appearing circular with irregular edges and measuring up to ~1000 µm in diameter. Safranin staining revealed higher color saturation in aggregates, reflecting increased biomass and EPS. Both morphologies were present in all samples, indicating incomplete disaggregation by sonication.

To evaluate BacQuant performance, images were treated as biological replicates and manually annotated to generate ground truth labels. There was a significant difference (p=0.0001; **Figure 1C** ) in the number of free cells identified by ground truth and those identified by BacQuant. This underestimation likely arises from pixel-based normalization to a user-defined average cell area, which does not account for cell orientation or morphological variability.

In contrast, BacQuant slightly overestimated aggregate counts compared to ground truth, although this difference was not statistically significant (p=0.0569; **Figure 1D** ). This trend likely reflects conservative edge detection during segmentation and the lack of depth information in two-dimensional images, which may obscure internal aggregate structure.

BacQuant total cell counts were compared to traditional VPC measurements obtained via serial dilution and drop-plating ( **Figure 1A, E).** Across twenty samples, BacQuant produced significantly higher mean cell counts than VPC (p=0.0001), with averages of 1.18 x 10 ^11^ and 4.46 x 10 ^7^ cells, respectively ( **Figure 1F** ). BacQuant results were more tightly clustered, whereas VPC values exhibited greater variability.

This discrepancy is consistent with the presence of biofilm aggregates that contain many cells but yield only a single colony on agar plates. Additionally, BacQuant includes dead cells in its estimates, whereas VPC counts only viable cells, further contributing to higher total counts.

This study introduces BacQuant, a computationally inexpensive image-processing pipeline for quantifying biofilm cells following sonication. BacQuant addresses a fundamental limitation of CFU-based methods by explicitly accounting for residual aggregates that persist after mechanical disruption and confound viable cell estimates (Fleming et al., 2020). By combining thresholding with edge and contour detection, BacQuant enables reproducible instant segmentation of individual cells and aggregates.

Sonication was selected as the representative disruption method due to its widespread use in laboratory, clinical, and industrial contexts. While sonication effectively detaches biofilms from surfaces, it does not reliably dissociate biofilms into single cells, leaving EPS-bound aggregates intact (Kragh et al., 2016) ( **Figure 1B** ). These aggregates are indistinguishable from single cells in VPC assays, leading to systematic underestimation of bacterial load. BacQuant circumvents this issue by estimating the number of cells within aggregates, producing significantly higher and more consistent counts than VPC ( **Figure 1F** ).

A major strength of BacQuant is its accessibility. Unlike machine learning-based or confocal approaches, the pipeline does not require training data, specialized hardware, or bulky software installations. It can be run on standard CPUs and adapted to diverse imaging conditions, making it broadly applicable across laboratories. The method captures biologically meaningful structure features, specifically the distinction between free cells and aggregates, that are routinely missed by culture-based techniques.

Nevertheless, BacQuant has limitations. In dense images, over-segmentation may occur, leading to slight overestimation of aggregate numbers ( **Figure 1D )** . Conversely, free cells are underestimated due to reliance on an average cell area parameter that does not account for morphological variability ( **Figure 1C ).** The pipeline also cannot distinguish between live and dead cells, which may inflate estimates relative to viable counts. If cells occur in small clusters like doublets or triplets, it is processed as an aggregate while they could be a few individual cells in close promixity to one another. Another major limitation is the imposition of 2D structure on the 3D aggregate morphology. Because BacQuant only uses two-dimensional estimation, edge detection is conservative and does not take the height of the structure into account when determining cell density within large cell clusters, dampening the true estimate of cell density within. Additionally, accurate performance depends on high-contrast microscopy images and correct calibration for each experimental setup.

Despite these constraints, BacQuant provides a rapid and scalable complement to traditional microbiological methods. The entire analysis can be completed within minutes, compared to hours or days required for plating assays. Accurate biofilm quantification is critical for antimicrobial testing, clinical research, and environmental monitoring, and BacQuant offers a practical tool for improving structural enumeration of bacterial populations (Folliero et al., 2021).

Future work should integrate live/dead staining, molecular viability markers, and three-dimension imaging to refine estimates of active biofilm populations (Sauer et al., 2022; Wang, Zhu, Zheng, Dong, & Liu, 2022). Incorporation of machine learning-based segmentation could further enhance classification of complex morphologies. Ultimately, BacQuant provides a foundation for automated, image-based biofilm quantification and highlights the value of computer vision approaches in microbiology research..

## Methods

**Materials and Methods**

*Bacterial Culture Preparation*

*P. aeruginosa* PAO1 liquid cultures were routinely grown in lysogeny broth (LB) (SigmaAldrich®, Cat# L3022) at 37°C under 220 RPM shaking conditions for 18 hours in 125 mL flasks.

*In vitro Well-plate Model*

*P. aeruginosa* biofilms were cultivated in 24-well non-tissue culture-treated plates (VWR, Cat# 10861-558) for 48 hours at 37°C under 80 RPM shaking conditions. Individual wells were inoculated with 10 ^6^ CFU/mL in 800 µL. After the 48-hour incubation, media was removed, and each well was rinsed with 1 mL of 0.85% saline solution to remove any unattached or lysed cells. This growth protocol was adapted from Fleming et. al (Fleming et al., 2020). The entire plate was covered with parafilm and placed into a water bath sonicator (Fischer Scientific, Cat# 15337411) for 30 minutes. This sonication condition was selected based on previously published biofilm disruption protocols (Fleming, Chahin, & Rumbaugh, 2017; Redman et al., 2021) and was not used independently optimized in the present study. Because the purpose of this work was to evaluate BacQuant as an image-based method for quantifying cells and residual aggregates following a standard disruption step, additional optimization of sonication intensity, during, or enzymatic pretreatment was outside the scope of this study. After sonication, the contents of each well were moved to a respective 1.5 mL microcentrifuge tube.

*Slide Preparation and Viability Assay*

After harvesting, 10 µL of cell solution was added onto a glass microscope slide (Globe Scientific, Inc, Cat# 1304). The solution was spread into a 1 cm ^2^ area at the center of each slide using an inoculation loop. The samples were then heat-fixed onto the slides and stained with Safranin Advanced Counterstain (Hardy Diagnostics, Cat# GK400) for 1 minute and subsequently rinsed with deionized water. The slides were then wet-mounted using immersion oil and topped with a glass cover slip. Concurrently, the remaining cell solution was serially diluted in saline and drop-plated onto solid LB agar and incubated for 18-24 hours at 37°C for CFU enumeration.

*Brightfield Microscopy*

All slides were imaged using an Olympus BX43 microscope under oil immersion at 100x magnification. Five representative images of each slide were taken, representing one well in the 24-well plate model. An Olympus DP22 camera was used with CellSens Entry software to take the images. The size of each image and light intensity was constant across all images. The dimensions of each image were measured to be 5000 x 7000 nm, in metric units using the Linear Ruler tool in CellSens Entry. Images were saved and label-matched to their corresponding drop-plate. 

*Image Analysis*

A custom computer vision image analysis pipeline was built using Python v3.8 programming language. A stand-alone version of the pipeline is provided and can be run as an online notebook in or as a script with a Python IDE. All necessary interaction with the code is clearly labeled for the user as comments in the code, as this pipeline is not based on a graphical user interface (GUI). This pipeline should be adapted for individual experiments, as it is currently optimized for use with *P. aeruginosa* mono-species biofilm cells. All parameters are clearly labeled in the code and are easily customizable. All code is freely available, with the implementation of each step of the pipeline clearly shown. The code is available through Google Colaboratory (https://colab.research.google.com/drive/1JWpmjoXKtgYmeux7MUyBH6syZLHm1inf?usp=sharing). We recommend this pipeline be used with GPU acceleration for high-throughput analyses, but running the process in the cloud or with a CPU is also easily supported. The pipeline takes *.png* file format images as the primary input, as well as user-specified parameters such as the area of an individual cell in pixels and image size.

The first step of the pipeline entails generating a binary mask of the image based on color threshold values. The mask eliminates background noise in the image and standardizes the shape and sizes of all image features. Following masking, a diameter-based size thresholding algorithm is used to label large connected regions in the image as aggregates in blue, and smaller regions as free cells in yellow. This simplifies the visual distinction between aggregates and free cells. From this color-changing step, the percentages of aggregated biomass and free cells are determined via color-based pixel pooling. For the purpose of this analysis, an aggregate is defined as any connected segmented region with an estimated diameter greater than the user-defined threshold for a single cell. Segmented regions at or below this threshold are classified as free cells. Smaller clusters like doublets, triplets, and quadruplets are classified according to the same size-based rules and were not assigned separate biological categories. If the connected region exceeds the aggregate threshold, it is labeled as an aggregate and its cell number is estimated by dividing the segmented area by the user defined single-cell average area in pixels. If the region does not exceed the threshold, it is classified as a free-cell region.

Concurrently, a contour detection algorithm from the OpenCV (https:// github.com/itseez/opencv ) library is used to extract aggregated regions of cells as features. The edges of each aggregate are separated from the background image using Canny edge detection (DOI: 10.1109/TPAMI.1986.4767851). Then, the remaining regions are enclosed by a boundary line through contour detection. This allows for the thorough definition of the 2D shape of each aggregate. From these contours, the area of each aggregate is calculated in pixels and converted to square microns. Cell counts within the aggregates are based on the area of an individual cell in pixels, which is defined by the user at the beginning of the pipeline. It is important to note that this process only works on two-dimensional images. It also can be used with epifluorescence microscopy and live/dead staining with modification in the image processing code to exclude dead cells. It is not optimized for Z-stack images, though it can be modified to work with image arrays and high-throughput imaging results. This makes BacQuant a useful tool for more basic laboratories without complex equipment, software, or imaging capabilities.

The output of this pipeline includes the percent aggregated biomass in each image, number of aggregates, estimated number of cells in aggregates, total estimated cells in each image, and estimated calculation of cells per mL of solution. The raw data from this analysis is stored as a *.csv* file format spreadsheet. We recommend that further processing of the data be conducted in Microsoft Excel.

*Statistical Analysis*

Statistical analysis was conducted using GraphPad Prism. A paired t-test was utilized to compare ground truth and BacQuant counts of aggregates and free cells. An unpaired t-test was used to compare viable plate count assay results with BacQuant results. All analyses were conducted to determine statistical significance with an alpha value of 0.05.

## References

[R1] Acosta Nicole, Waddell Barbara, Heirali Alya, Somayaji Ranjani, Surette Michael G., Workentine Matthew L., Rabin Harvey R., Parkins Michael D. (2020). Cystic Fibrosis Patients Infected With Epidemic Pseudomonas aeruginosa Strains Have Unique Microbial Communities. Frontiers in Cellular and Infection Microbiology.

[R2] Beal Jacob, Farny Natalie G., Haddock-Angelli Traci, Selvarajah Vinoo, Baldwin Geoff S., Buckley-Taylor Russell, Gershater Markus, Kiga Daisuke, Marken John, Sanchania Vishal, Sison Abigail, Workman Christopher T., Pehlivan Meryem, Roige Biel Badia, Aarnio Tiu, Kivisto Samu, Koski Jessica, Lehtonen Leevi, Pezzutto Denise, Rautanen Pauliina, Bian Weixin, Hu Zhiyuan, Liu Zhihao, Liu Zi, Ma Liang, Pan Luyao, Qin Zichen, Wang Huichao, Wang Xiangxuan, Xu Hao, Xu Xia, El Moubayed Yorgo, Dong Shan, Fang Choco, He Hanker, He Henry, Huang Fangliang, Shi Ruyi, Tang Cassie, Tang Christian, Xu Shirly, Yan Calvin, Bartzoka Natalia, Kanata Eleni, Kapsokefalou Maria, Katopodi Xanthi-Leda, Kostadima Eleni, Kostopoulos Ioannis V., Kotzastratis Stylianos, Koutelidakis Antonios E., Krokos Vasilios, Litsa Maria, Ntekas Ioannis, Spatharas Panagiotis, Tsitsilonis Ourania E., Zerva Anastasia, Annem Vidhya, Cone Eli, Elias Noel, Gupta Shreya, Lam Kendrick, Tutuianu Anna, Mishler Dennis M., Toro Bibiana, Akinfenwa Akinwumi, Burns Frank, Herbert Heydy, Jones Melissa, Laun Sarah, Morrison Shikei, Smith Zion, Peng Zhao, Ziwei Zhou, Deng Rui, Huang Yilin, Li Tingyue, Ma Yingqi, Shen Zhiyuan, Wang Chenxi, Wang Yuyao, Zhao Tianyan, Lang Yusen, Liang Yuteng, Wang Xueyao, Wu Yi, Aizik Dror, Angel Sagi, Farhi Einan, Keidar Nitzan, Oser Eden, Pasi Mor, Kalinowski Jorn, Otto Matthias, Ruhnau Johannes, Cubukcu Hande, Hoskan Mehmet Ali, Senyuz Ilayda, Chi Jordi, Sauter Antoni Planas, Simona Magda Faijes, Byun Sumin, Cho Sungwoo, Kim Goeun, Lee Yeonjae, Lim Sangwu, Yang Hanyeol, Xin Tian, Yaxi Zhang, Zhao Peng, Han Weitang, He Fa, He Yuna, Li Nuonan, Luo Xiaofan, Boxuan Cheng, Jiaqi Hu, Liangjian Yang, Wanji Li, Xinguang Chen, Xinyu Liu, Wu Zishi, Xi Yukun, Yang Xilin, Yang Yuchen, Yang Zhuoyi, Zhang Yihao, Zhou Yuezhang, Peng Yue, Yadi Liu, Yang Shaobo, Yuanxu Jiang, Zhang Kecheng, Abraham Doris, Heger Theresa, Leach Cass, Lorch Kevin, Luo Linda, Gaudi Alex, Ho Anthony, Huang Morris, Kim Christine, Kugathasan Luxcia, Lam Kevin, Pan Catherine, Qi Ariel, Yan Cathy, Schaaf Kaitlin, Sillner Cassandra, Coates Ryan, Elliott Hannah, Heath Emily, McShane Evie, Parry Geraint, Tariq Ali, Thomas Sophie, Chen Ching-Wei, Cheng Yu-Hong, Hsu Chia-Wei, Liao Chin-Hsuan, Liu Wei-Ting, Tang Yu-Cheng, Tang Yu-Hsin, Yang Zon En, Jian Liu, Li Caidian, Lin Chenyi, Ran Guozheng, Run Zhouyan, Ting Weiyu, Yong Zhangxiang, Yu Liuhong, Lind Andrea Clausen, Norberg Axel, Olmin Amanda, Sjolin Jacob, Torell Agnes, Trivellin Cecilia, Zorrilla Francisco, Vries Philip Gorter de, Cheng Haolun, Peng Jiarong, Xiong Zhenyu, Altarawneh Dina, Amir Sayeda Sakina, Hassan Sondoss, Vincent Annette, Costa Ben, Gallegos Isabella, Hale Mitch, Sonnier Matt, Whalen Kathleen, Elikan Max, Kim Sean, You Jaewon, Rambhatla Rahul, Viswanathan Ashwin, Tian Hong, Xu Huandi, Zhang Wanli, Zhou Shuyao, Jiamiao Liu, Jiaqi Xiao, Craw Darilyn, Goetz Marley, Rettedal Neil, Yarbrough Hayden, Ahlgren Christopher, Guadagnino Brett, Guenther James, Huynh Juilanne, He Zhien, Liu Huan, Liu Yuansheng, Qu Mingbo, Song Li, Yang Chao, Yang Jun, Yin Xianqi, Zhang Yuanzhen, Zhou Jianan, Zi Lihan, Jinyu Zhu, Kang Xu, Xilei Peng, Xue Han, Xun Shu, Babu Priyanka, Dogra Arushi, Thokachichu Pranav, Faurdal David, Jensen Joen Haahr, Mejlsted Jacob, Nielsen Lina, Rasmussen Tenna, Denter Jennifer, Husnatter Kai, Longo Ylenia, Luzuriaga Juan Carlos, Moncayo Eduardo, Moreira Natalia Torres, Tapia Jennifer, Dingyue Tang, Jingjing Zhao, Wenhao Xu, Xinyu Teng, Xiujing Hong, DeKloe Jackson, Astles Ben, Baronaite Ugne, Grazulyte Inga, Hwang Michael, Pang Yibo, Crone Michael Andrew, Hosseini Reza, Houmani Moustafa, Zadeh Daniel, Zanotti Violetta, Baltensperger Oliver Andreas, Bijman Eline Yafele, Garulli Elisa, Krusemann Jan Lukas, Martinelli Adriano, Martinez Antonio, Vornholt Tobias, Camille Monteil, Paul Ahavi, Browne Emily, Gilman Daniel Barber James, Hewitt Amy, Hodson Sophie, Holmedal Ingebjorg, Kennedy Fiona, Sackey Juliana, Beck Selina, Eidloth Franziska, Imgold Markus, Matheis Anna, Meerbrei Tanja, Ruscher David, Schaeftlein Marco, Hanrong Zhu, Wan Mitchell, Dai Leijie, Jin Kaifeng, Wang Sihan, Wang Xin, Wang Yi, Wang Yifan, Wu Chenhai, Zhang Zixuan, Zhou Yineng, Xinyu Liu, Zirong Zeng, Babar Rehmat, Brewer Mathew, Clodomir Christina, Das Neves Laura, Iwuogo Amanda, Jones Ari, Jones Cara, Kelly Julia, Kim Gloria, Siemer Jessica, Yadav Yash, Ikagawa Yuichiro, Isogai Tatsuki, Niwa Ryo, Aubry Celine, Briand William, Jacq Annick, Lautru Sylvie, Marta Britany, Maupu Clemence, Ollessa-Daragon Xavier, Papadopoulo Kenn, Azad Mahnaz Sabeta, Kuangyi Wei, Xiu Yao, Yang Chenghao, Iyer Aditya, Prins Rianne, Yesley Phillip, Lichi Fang, Xuan Chen Zi, Jo Kyuhee, Park Mikyung, Park Seunghyun, Yoo Hojun, Burckhardt Nele, Daniels Lea, Klopprogge Bjarne, Kruger Dustin, Meyfarth Oda-Emilia, Putthoff Lisa, Wawrzyniak Dominika, Hu Xinyi, Wang Yunyi, Badash Lior, Baichman-Kass Amichai, Barshap Alon, Friedman Yonatan, Milshtein Eliya, Vardi Omri, Dong Shan, Gu Yining, Pei Yuanzhe, Shi Ruyi, Yang Fan, Yang Jinshu, Zhu Xueqian, Ching Lam Kai, Ching Law Hiu, Chun Ng Tsz, Hin Yu Man, Hong Lai Tsz, Lam Chan Wing, Lam Yiu Choi, Matthew Cheah, Ngo Cheng Tsz, Shuan Yun, Wan Chan Tsey, Yan Tsui Shing, Yee Chong Yuk, Yu Tam Chi, Yu Yuen Wai, Anson Chung Tsun Ho, Choi Lee Sze, Chun Cheung Man, Hin Chan Lok, Hin Wong Chung, Ho Ng Sze, Jay Leung Chung Yin, Katherine Lai Man Wai, Kin-ning Wong Carol, Kiu Lee Hong, Kong Cheng Chak, Wai Leung Chung, Yan Yeung Wing, Yeung Wong Tsz, Yin Lee Ka, Grace Tsui Shing Yan, Joe Lam Kai Ching, Kenneth Ng Tsz Chun, Shuan Cheah Matthew Yun, Aldo Ferdinan, Pang Chung Him, So Kam Pang, Wong Hei Man, Ching Lai Tsz, Ching Luk Hau, Fung Ip Ning, Fung Yam Shing, Hong Lee Chi, Ning Hsiu Ou, Sang Jonathan Cheng Hon, Elsa Yeung Hoi Lam, Hei Chan Yick, Sing Lo Ho, Wang Choi Seong, Gu Yiheng, Rong Ziyue, Song Haoyue, Wang Pengying, Wang Yuefei, Chen Yan, Qiu Hao, Ren Haotian, Xiao Ziyang, Heng Heng, Rao Xichen, Tian Ruonan, Deb Shalini S., Kamble Yash Laxman, Kumbhojkar Ninad, Patel Bhargav, Prakash Supriya, Reshamwala Shamlan M. S., Taskar Poorva, Gokul, Uday Adwaith B., Basu Anubhav, Gandhi Rishi, Khaimani Jatin, Khenwar Arundhati, Raut Sandeep, Somvanshi Tejas, Das Diptatanu, Ghosh Souvik, Rai Hrishika, Anand Nithishwer Mouroug, Jainarayanan Ashwin Kumar, Kalson Pranshu, Liya Devang Haresh, Mishra Vibhu, Pai Sveekruth Sheshagiri, Pitaliya Madhav, Rana Yash, Yadav Ravineet, Arora Neha, Arora Vasu, Jain Shubham, Patel Abhilash, Sharma Saksham, Singh Priyanka, Goenka Anushya, Jain Rishabh, Jha Aryaman, Kumar Adarsh, Soni Abhinav, Ananthakrishnan Sathvik, Devi Velvizhi, Faidh Mohammed, Jayaraman Guhan, Kittur M. Sagar, Mahapatra Nitish R., Menon Sarvesh, Muthukrishnan Anantha Barathi, Kailash B. P., Sabuwala Burhanuddin, Shinde Mousami, Venkatraghavan Sankalpa, Liu Weijia, Miao Zhoudi, Wang Tian, Wang Yaling, Zhang Shuyan, Chai Ruochen, Ge Yubin, Hou Ali, Liu Fangqi, Liu Xutong, Mao Jiangjiao, Wang Zihao, Yu Haimeng, Yuan Hetian, Zhan Yang, Ries Anna, Wolfbeisz Chiara, Kanaya Toshihiro, Kawasaki Yusuke, Maruo Tatuya, Mori Yuya, Satoh Takehito, Chau Anthony, Chu Wai Yan, Markiv Anatoliy, Marti Marcos Vega-Hazas, Medina Maria Jose Ramos, Raju Deeksha, Sinha Shubhankar, Choi Youngeun, Ryu Bo Sun, Byagathvalli Gaurav, Kim Ellie, Crooijmans Marjolein, Waard Jazzy de, Amstel Chiel van, Demchuk Aubrey, Haight Travis, Kim Dong Ju, Neda Andrei, Roberts Luc, Saville Luke, Takeyasu Reanna, Tobin David, Akbary Mina, Avileli Rebecca, He Karen, Pageni Aroma, Saville Luke, Silva Dewuni De, De Silva Nimaya, Turton Kristi, Wu Michelle, Zhang Alice, Chavez Benjamin, Garavito Paula, Latham Michael, Ptak Jeffrey, Tharp Darron, Izzati Nurul, Jonsson Martin, Labecka Nikol, Palo Sara, Beale Renee, Logel Dominic, Mellou Areti-Efremia, Myers Karl, Alonso Alejandro, Cifuentes Rodrigo Hernandez, Clemente Borja Sanchez, Gonzalo Gonzalo Saiz, Hernandez Ivan Martin, Hernandez Laura Armero, Lombardero Francisco Javier Quero, Marquina Domingo, Rodriguez Guillermo Fernandez, Smet Ignacio Albert, Butterfield Tom, Deshmukh-Reeves Ed, Gogineni Namrata, Hemmings Sam, Kabbara Ismat, Norvaisaite Ieva, Smith Ryan, Bauersachs Daniel, Daniel Benjamin, Inckemann Rene, Seiffermann Alexandra, Stukenberg Daniel, Weile Carl, Clerc Valerian, Ha Jacqueline, Totten Stephanie, Chang Thomas, Jimenez Carlene, Maddiboina Dhanyasri, Acar Beliz Leyla, Elcin Evrim, Inanc Tugba, Kantas Gamze, Kayihan Ceyhun, Secen Mert, Suer Gun, Ucan Kutay, Unal Tunc, Fischer Matthew, Jasti Naveen, Stewart Thomas, Caldwell Sarah, Lee Jordan, Schultz Jessica, Chang Ting-Chen, Chen Pei-Hong, Cheng Yu-Hsuan, Hsu Yi-Hsuan, Yeh Chan-yu, Ding Zhipeng, Li Zihao, Lockwood Savannah, Quinn Katherine, Carrillo Leo, Heintze Maxime, Meneu Lea, Peras Marie, Yehouessi Tamara, Eilers Keno, Falgenhauer Elisabeth, Kiu Wong Hoi, Mayer Julia, Mueller Julia, von Schoenberg Sophie, Schwarz Dominic, Tunaj Brigit, Hu Zhaoqing, Huang Yansong, Li Yuanyuan, Fang Chengzhu, Liu Jiangyuan, Liu Yiheng, Wu Yaxuan, Xu Sheng, Yuan Long, Edelmayer Marco, Hiesinger Marlene, Hofer Sebastian, Krainer Birgit, Oswald Andreas, Strasser Dominik, Zimmermann Andreas, Chen Yi-Cian, Chan Yuan-Yao, Chang Yu-Ci, Deng Nian Ruei, Ku Chi-Yao, Lee Meng-Zhan, Li Hailong, Liu Zhaoyu, Song Guowei, Xiang Yuening, Yan Hongfa, Huanying He, Qiaochu Jiang, Shengjuan Jiang, Yujie Peng, Burridge Matt, Standforth Kyle, Went Sam, Chenxi Liang, Han Wang, Qipeng Zhang, Yifan Li, Yiming Quan, Yutong Pan, Kou Senhao, Luan Lin, Akova Umut, Fitzgerald Liza, Ikwuagwu Bon, Johnson Michael, Kurian Jacob, Throsberg Christian, Allen Lucy, Humphreys Christopher, Partridge Daniel, Whittle Michaella, Zilinskaite Nemira, Lee Meixuan, Lin Weifeng, Ma Yuan, Wang Kai, Cheng Hsuan, Chi Shumei, Chuang Yi-Chien, Huang Ray, Ko LiangYu, Lin Yu-Chun, Tsai You-Yang, Wang Cheng-Chieh, Yu Kai-Chiang, Burud Hanna Nedreberg, Chen Carmen, Haralsvik Anne Kristin, Marinovic Adrian, Pedersen Hege Hetland, Sande Amanda, Solvang Vanessa, Ming Shaw Kar, Praditya Albert, Abduraimova Aiganym, Meirkhanova Ayagoz, Mukhanova Assel, Mulikova Tomiris, Gou Yanchen, Lu Chenyu, Ma Jiaxin, Zhu Chushu, Aaron Leow Chung Yong, Iyer Tvarita Shivakumar, Jiacheng Wu, Lim Yan Ping, Lin Beatrix Tung Xue, Ramzeen Aaron, Yong Nur Liyana Binte Ayub Ow, Chua Yah Tse Sabrina, Fong Yuhui Deborah, He Menglan, Tan Li Yang, Jiahe Zhang, Mingge Li, Nianlong Li, Yueyi Li, Yuhan Cheng, Chang Annabel, Chen Chih-Chiang, Chou Ryan, Clapper Jude, Lai Evelyn, Lin Yasmin, Wang Kelsey, Yang Jake, Anwar Mariam, Chehade Ibrahim, Hariyani Imtiyaz, Hau Sion, Isaac Ashley, Karpauskaite Laura, Magzoub Mazin, Obaji Daniel, Song Yong Rafael, Yun Yejie, Sun Kai, Zhang Yunqian, Beard Eleanor, Crosby Laurel Constanti, Delalez Nicolas, Karshenas Arman, Kozhevnikov Adrian, Kryukova Jhanna, Saini Karandip, Stocks Jon, Sudarshan Bhuvana, Taylor Max, Wadhams George, Windo Joe, Ameziane Annissa, Bhatt Darshak, Casas Alexis, Levrier Antoine, Santos Ana, Sia Nympha Elisa M., Wintermute Edwin, Dejoux Alice, Gopaul Deshmukh, Guerassimoff Lea, Jaoui Samuel, Madelenat Manon, Petracchini Serena, Cai Fu, Jianzhao Yang, Shuyu Shi, Tairan Li, Xin Li, Yongjie Lin, Zhecheng Huang, Becker Evan, Greenwald Matthew, Hu Vivian, Pavelek Tucker, Pinto Elizabeth, Wei Zemeng, Burgland Zachary, Chan Janice, Dejoie Julianne, Fitzgerald Kevin, Hartley Zach, Rasheed Moiz, Schacht Makayla, Gahagan Maddison, Kelly Ellis, Krauss Elisha, Cao Yuze, Shen Yishen, Wang Xuan, Xu Hanning, Zhang Jianxiang, Chandramouli Priyanka, Ashwin F Amal Jude, Jayaraman Srimathi, Jude Marcia Smiti, Kumar Vignesh, Lekshmi Hema, Preetha R., Rashid Khadija, Deepak Kumar S., Mohan Kumar B. S., Barrat Leon Michael, Dissmann Jil-Sophie, Kalinowski Jorn, Otto Matthias, Ruhnau Johannes, Stuhlweissenburg Fynn, Ueding Elisa, Bohner Ariel, Clark Brittany, Deibel Emilie, Klaas Liz, Pate Kaylee, Weber Elisa, Cohen Katherine, Guseva Anna, King Stefanie, Yoon Soohyun, Ambre Sanika, Bhowmick Shilpa, Pange Nishtha, Parab Komal, Patel Vainav, Patil Mitali, Rajurkar Aishwarya, Rege Mayuri, Sawant Maithili, Sawant Shrutika, Vaidya Anjali, Ji Peicheng, Luo Fang, Ma Guanghui, Xu Xin, Yin Jiacheng, Zhou Yinchi, Zhu Ke, Huang Yaohua, Huang Yinpin, Li Jiadong, Li Xuecheng, Wang Hao, Wang Ken, Wang Wei, Zhang Xinyu, Zou Jiahua, Bao Minyue, Kang Han, Liu Xiaolong, Tao Yibing, Wang Ziru, Yang Fuqiang, Zhang Tianyi, Zhong Yanling, Liu Jiezheng, Liu Jingang, Ma Lingling, Niu Xubo, Qian Ling, Wang Li, Yan Qingyan, Zhao Nannan, Chen Weixuan, Zhou Yuxin, Chen Junyang, Hao Junyao, HuaYue Zhang, Li Peilin, Pei Yifei, Qu Jingting, Wang Raven, Wang Xinyue, Wu Kangjie, Wu Yuxuan, Xiang Meredith, Yang Leyi, Yang Zisang, Zhaoting Li, Fu Wenhan, Li Zonghao, Tang Weiyi, Zhang Kaida, Li Haocong, Shao Xuze, Yang Chuyi, Zeng Yuanhong, Zhou Yanjun, Dong Shangzhi, Jung Younji, Li Sophie Ruojia, Li Tingting, Yu Jiacheng, Dong Shangzhi, Li Tingting, Miao Xinyi, Wang Sibo, Ding Yiming, Huang Jiaxi, Li Yuqi, Sun Ting, Tian Qinghe, Wu Mengxuan, Xing Jinming, Xiong Xin, Yan Yining, Yihang Qiu, Zhang Jige, Zhou Yi, Zhou Zhiyu, Chen Zhuoyang, He Peixiang, Hong Yirui, Hsiao Chia-Yi, Liang Zhihan, Liu Zhixiang, Ran Yuncong, Sun Shiyu, Xia Ruoyu, Dong Dongyang, Zhao Wenxue, Hu Miao, Hu Shi, Shi Wei, Shulun, Yan Han, Ye Yusheng, Hong Yiquan, Pan Yuyao, Song Yiran, Zhang Jinhan, Zhao Yihang, Chater Dounia, Foda Asmaa, Li Yanyan, Saade Ursula, Sayous Victor, Mo Yilin, Ren Wenan, Zeng Chenxu, Cao Yixin, Czekster Clarissa, Dunstan Izzy, Powis Simon, Reaney Bethany, Snaith Eva, Young Cam, Frankel Eva, Glockner Eleanor, Justice Isaac, Murugan Santosh, Penny Leo, Garcia Chrismar, Rentouli Stamatina, Aggarwal Priya, Budhan Stephanie, Chiang Woody, Kwasniak Dominika, Ledalla Karthik, Lee Matthew, Lo Natalie, Mullin Matthew, Pan Lin Yu, Rakhimov Jennifer, Ruzic Robert, Shah Manvi, Velikov Lukas, Vincent Sara, Horz Philip, Kuebler Nadine, Notheisen Jan, Doyle David, Gu Jiajun, Hu Wenyue, Yang Shuting, Kehan Tao, Menghan Gao, Xiaowen Mao, Chen Yifei, Kang Ziqi, Ni Haochen, Chen Junyu, He Lindong, Luo Mingyue, Tang Jiaqi, Boyce Kira, Lee James, Martin Michael, Nguyen Judy Van, Wan Leon, Astapenka Artur, Badalli Turan, Borovko Irina, Chulkova Nadezhda, Faustova Ilona, Kolosova Anastasia, Loog Mart, Maljavin Artemi, Matiyevskaya Frida, Tuzov Vladislav, Chang Catherine, Chou Ryan, Clapper Jude, Ho Tim, Hsieh Yi Da, Lai Evelyn, Tsai Leona, Wang Kelsey, Wu Justin, Dominguez Viana Isabel Perez, Fernandez Cesar Ibrahym Rodriguez, Fierro Daniela Olono, Nunez Anna Karen Aguilar, Perez Jose Pablo Rascon, Rivera Mario Loya, Trevizo Cynthia Lizeth Gonzalez, Velasco Maria Antonia Luna, Oropeza Carlos Javier Cordero, Mendoza Adrian Federico Hernandez, Figueroa Jose Arnulfo Juarez, Leal Luis Mario, Benavides Samantha Ayde Pena, Martinez Victor Javier Robledo, Aguirre Adriana Lizeth Rubio, Alvarado Andres Benjamin Sanchez, Solano Margarita Sofia Calixto, Castillo Nora Esther Torres, Zamora Alejandro Robles, Pena Thevenet Esteban de la, Blas Karla Soto, Huerta Ana Laura Torres, Resendiz Armando Cortes, Cruz Frida, Diaz Fernanda, Espinoza Diego, Figueroa Ana Cristina, Luque Ana Cecilia, Portillo Roberto, Senes Carolina, Tamayo Diana, Del Toro Mariano, Alexopoulos Ioannis, Giannopoulos Alexandros Dimitriou, Giannoula Yvoni, Kyrpizidis Grigorios, Xinyue Ma, Xirui Chen, Zhiwei Song, Adler Nina, Caballero Amalia, Hamady Carla, Ibrahim Ahmed, Parmar Jasmeen, Rastogi Tashi, Yang Jindian, Delhomme Jean, Henras Anthony, Heux Stephanie, Romeo Yves, Toanen Marion, Wagner Camille, Zanoni Paul, Lotz Thea, Nickels Elena, Suss Beatrix, Warzecha Heribert, Zimmermann Jennifer, Dubuc Emilien, Eijkens Bruno, Keij Sander, Twisk Simone, Verhagen Mick, Oetelaar Maxime van den, Armstrong Alexander, Bennis Nicole, Bouwmeester Susan, Buller Lisa, Kohabir Kavish, Leeuw Monique de, Mangkusaputra Venda, Mattens Jard, Nijenhuis Janine, Paez Timmy, Schmidtchen Lisbeth, Voort Gemma van der, Ge Gao, Haoran Xu, Xiaojin Li, Agena Ejouan, Agena Ethan, Bath Scott, Campbell Robert, Dalangin Rochelin, Kim Anna, Sauvageau Dominic, Shkolnikov Irene, Graves Daniel, Lang Jacob, Nieberding-Swanberg Jolee, Rao Achala, Torres Ares, Yao Andrew, Abbas Anser, Luo Claire, Zepeng Xu, Ziyi Zhao, Chen Janice, Colgan Cian, Dvorkin Steve, Filzen Rachael, Patel Varun, Scott Allison, Zulueta Patricia, Acosta Joaquin, Araya Lucas, Chavez Francisco, Farias Sebastian, Garrido Delia, Marcoleta Andres, Munoz Felipe, Rivas Paula, Colant Noelle, Fan Catherine, Frank Stefanie, Gabrielli Jacopo, Handal Paola, Pinheiro Vitor, Santamaria Stefanie, Withanage Shamal, Xue Fang, Gerard Antoine, Lefevre Marine, Milano Fiona, Oliveira Nina De Sousa, Parmentier Mathieu, Rigon Luca, Chamiec-Case Elizabeth, Chen Ryan, Crowley Peter, Doyle Shannon, Kadimi Sricharan, Vella Toni, Adulyanukosol Natthawut, Dusseaux Theodore A., Forman Victor, Hansen Cecilie, Kofoed Selma, Louis Simon, Lykkegaard Magnus Ronne, Mancinotti Davide, Meyer Lasse, Michelsen Stephanie, Raadam Morten, Rasmussen Victoria Svaerke, Thormar Eirikur Andri, Uslu Attila, leelahakorn Nattawut, Ding Shizhi, Li Changyu, Tan Huishuang, Xu Yinsong, Yang Jianzhe, Gamoneda Diego, Kantor Nicole, Trujillo-Rodriguez Lidimarie, Turner Matthew, George Stephan, McConnell Kelton, Pollitt Chynna, Amin Ihya Fakhrurizal, Ikhsan Muhammad, Japranata Valdi Ven, Laurentius Andrea, Nurachman Luthfian Aby, Pratama Muhammad Iqbal Adi, Dirven Yvette, Frohlich Lisa, Linke Dirk, Mertes Verena, Rolfsnes Rebekka Rekkedal, Saragliadis Athanasios, Castillo Sandra, Chinnathambi Sathivel, Ellermeier Craig, Farrell Jennifer, Fassler Jan, Fuentes Ernie, Ryan Sean, Sander Edward, Bott Amie, Healy Liam, Karumanchi Pranathi, Ruzicka Alex, Wang Ziyu, Byatt Gabriel, Despres Philippe C., Dube Alexandre, Lemieux Pascale, Echelard Florian, Lortie Louis-Andre, Rouleau Francois D., Barrington Seth, Basulto Cynthia, Delgadillo Sabrina, Leon Karen De, Madrid Micah, Meas Catherosette, Sabandal Angelica, Sanchez Magaly Aguirre, Tsui Jennifer, Woodward Noble, Battina Rohith, Boyer Jess, Cherupalla Arjun, Chiang Jason, Heng Mary, Keating Collin, Liang Tommy, Loke Chun Kit, Premo Jacob, Srinivasan Keerthana, Starkel John, Zheng Daniel, Astorino Gabe, Cott Rachel Van, Guo Jintao, Kortus Drew, Niu Wei, Freire Paulo J. C., Pedrolli Danielle Biscaro, Ribeiro Nathan Vinicius, Silva Bruna Fernandes, Vanini Nadine Vaz, Mota Mariana Santana da, Crispim Larissa de Souza, Chapman Tyler, Gaitt Tobias, Jones Megan, Watson Emily, Lopez-Grado Guillem, Sans Laura, Brink Matilda, Rajagopal Varshni, Ramstrom Elin, Bete Anna, Camacho Yazmin, Carter Jonah, Davis Christina, Dong Jason, Ehrenworth Amy, Goodson Michael, Guptil Chris, Herrmann Max, Hung Chia, Jesse Hayley, Krabacher Rachel, McDonald Dallas, Menart Peter, O’Leary Travis, Polanka Laura, Poole Andrea, Varaljay Vanessa, Appel Alana, Cave John, Huuki Liz, McDonough Matt, Mills Channah, Mitropoulos Alex, Pruneski James, Wickiser Ken, Buson Felipe Xavier, Flores Vinicius, Lima Guilherme Meira, Rosa Caio Gomes Tavares, Bao Guanke, Dong Haitao, Luo Zhi, Peng Jiarong, An Yongyan, Cheng Cheng, Jiang Zhenyu, Kong Linzhen, Luo Chenfei, Luo Liudong, Shi Yingying, Tang Erting, Wang Ping, Wang Yuyang, Xu Guiyang, Yu Wenfei, Zhang Bonan, Zhang Qian, Garcia David, Jiang Nannan, Kristy Brandon, Laurel Ralph, Leitner Karl, Loeffler Frank, Ripp Steven, Street Morgan, Amheine Khadija, Bindt Felix, Boer Meine, Boxem Mike, Govers Jolijn, Jongkees Seino, Pattiradjawane Lorenzo, Swart Pim, Tsang Helen, Graaf Floor de, ten Dam Marjolijn, Heijningen Franca van, Boada Yadira, Vignoni Alejandro, Brasas Valentas, Gaizauskaite Aukse, Jakutis Gabrielius, Jasiunas Simas, Juskaite Ieva, Ritmejeris Justas, Vaitkus Dovydas, Venclovas Tomas, Vitkute Kornelija, Yeliseyeva Hanna, Zukauskaite Kristina, Zvirblyte Justina, Karpus Laurynas, Mazelis Ignas, Rokaitis Irmantas, Akingbesote Ngozi D., Culfogienis Dylan, Huang William, Park Kevin, Ahuja Janvi, Corre Christophe, Dhaliwal Gurpreet, Evans Rhys, Hill Kurt, Holman Olivor, Jaramillo Alfonso, Khalid Alizah, Lawrence Jack, Mansfield Laura, O’Brien James, Ong June, Prakash Satya, Whiteside Jonny, Anderson Karl, Chun Emily, Kim Grace, Nguyen Aerilynn, Shola Chemay, Toghani Dorsa, Wong Angel, Wong Joanne, Yung Jay, Johnson Elizabeth, Lahad Divangana, Nicholson Kyle, Pedamallu Havisha, Phelan Cam, Fikry Clara, Fulton Leah, Lassel Nicole, Perera Dylan, Robin Marina, Shaw Nicolette, Bowman Kyle, Coleman Sarah, Emilov Kristian, Gaspar Camila, Jenakendran Jenaagan, Mubeen Sara, Obrvan Marko, Smith Caroline, Bo Tang, Du Liaoqi, Tianyi Chang, Yuan Xing, Yue Qing, Do Stephanie, Fang Xiangyi, Jones Ethan, Laury Jessica, Liu Wukun, Oliver Adam, Parr Lillian, Saha Margaret, Shen Chengwu, Son Tinh, Urban Julia, Verma Yashna, Zhou Hanmi, Dong Shan, Hao Zhengguo, Kuang Yi, Liu Ting, Zhou Rui, Arruda Beck, Farny Natalie, Hao Mei, Pearce Camille, Rebello Alex, Sharma Arth, Sumner Kylie, Sweet Bailey, Lin Junliang, Mengtao Du, Peiyao Fan, Xinlei Fang, Cai Niangui, Chen Junhong, Fu Yousi, Hu Yunyun, Qiang Ye, Wang Qiupeng, Yang Ruofan, Yucheng Chen, Zheng Jiyang, Chang Kevin, Gao Cecily, Isaacs Farren, Li Kevin, Moscoso Ricardo, Patel Jaymin, Telesz Lauren, Tirad Alice, Cao Qinhao, Feng Xinhua, Lu Yinjing, Zhang Xianyin, Zhou Xuanhao, Sun Dongchang, Yuan Zhe, Zhou Jiajie, iGEM Interlab Study Contributors, Aachen, Aalto-Helsinki, AHUT_China, Aix-Marseille, ASTWS-China, Athens, Austin_LASA, Austin_UTexas, Baltimore_BioCrew, BCU, BFSUICC-China, BGIC-Global, BGU_Israel, Bielefeld-CeBiTec, Bilkent-UNAMBG, BioIQS-Barcelona, BioMarvel, BIT, BIT-China, BJRS_China, BNDS_CHINA, BNU-China, BOKU-Vienna, BostonU, British_Columbia, Calgary, Cardiff_Wales, CCU_Taiwan, CDHSU-CHINA, Chalmers-Gothenburg, CIEI-BJ, CMUQ, CO_Mines, ColumbiaNYC, Cornell, CPU_CHINA, CSU_CHINA, CSU_Fort_Collins, Delgado-Ivy-Marin, DLUT_China, DLUT_China_B, DNHS_SanDiego, DTU-Denmark, Duesseldorf, Ecuador, ECUST, Edinburgh_OG, Edinburgh_UG, Emory, EPFL, ETH_Zurich, Evry_Paris-Saclay, Exeter, FAU_Erlangen, FJNU-China, Fudan, Fudan-CHINA, GDSYZX, Georgia_State, Gifu, GO_Paris-Saclay, GreatBay_China, Groningen, GZHS-United, HAFS, Hamburg, HBUT-China, HebrewU, HFLS_ZhejiangUnited, HK_HCY_LFC, HKJS_S, Hong_Kong_HKU, Hong_Kong_HKUST, Hong_Kong_JSS, Hong_Kong-CUHK, HUBU-Wuhan, HUST-China, HZAU-China, ICT-Mumbai, IISc-Bangalore, IISER-Bhopal-India, IISER-Kolkata, IISER-Mohali, IIT_Delhi, IIT_Kanpur, IIT-Madras, Jiangnan_China, Jilin_China, JMU_Wuerzburg, KAIT_JAPAN, KCL_UK, KUAS_Korea, Lambert_GA, Leiden, Lethbridge, Lethbridge_HS, Lubbock_TTU, Lund, Macquarie_Australia, Madrid-OLM, Manchester, Marburg, McGill, McMaster, METU_HS_Ankara, Michigan, MichiganState, Mingdao, Minnesota, Montpellier, Munich, Nanjing-China, NAU-CHINA, NAWI_Graz, NCHU_Taichung, NCTU_Formosa, NEU_China_A, NEU_China_B, Newcastle, NJU-China, NKU_CHINA, Northwestern, Nottingham, NPU-China, NTHU_Formosa, NTHU_Taiwan, NTNU_Trondheim, NTU-Singapore, NU_Kazakhstan, NUDT_CHINA, NUS_Singapore-A, NUS_Singapore-Sci, NWU-China, NYMU-Taipei, NYU_Abu_Dhabi, OUC-China, Oxford, Paris_Bettencourt, Pasteur_Paris, Peking, Pittsburgh, Purdue, Queens_Canada, RDFZ-China, REC-CHENNAI, Rheda_Bielefeld, RHIT, Rice, Ruia-Mumbai, SBS_SH_112144, SCAU-China, SCU-China, SCUT_ChinaB, SCUT-ChinaA, SDU-CHINA, SFLS_Shenzhen, ShanghaiTech, SHSBNU_China, SHSID_China, SHSU_China, SIAT-SCIE, SJTU-BioX-Shanghai, SKLMT-China, SMMU-China, SMS_Shenzhen, Sorbonne_U_Paris, SSHS-Shenzhen, SSTi-SZGD, St_Andrews, Stanford, Stanford-Brown-RISD, Stockholm, Stony_Brook, Stuttgart, SUIS_Shanghai, SYSU-CHINA, SYSU-Software, SZU-China, Tacoma_RAINmakers, Tartu_TUIT, TAS_Taipei, Tec-Chihuahua, Tec-Monterrey, TecCEM, TecMonterrey_GDL, Thessaloniki, Tongji_China, Toronto, Toulouse-INSA-UPS, TU_Darmstadt, TU-Eindhoven, TUDelft, TUST_China, UAlberta, UC_Davis, UC_San_Diego, UCAS-China, UChicago, UChile_Biotec, UCL, UCLouvain, UConn, UCopenhagen, UESTC-China, UFlorida, UGA, UI_Indonesia, UiOslo_Norway, UIOWA, UIUC_Illinois, ULaval, ULaVerne_Collab, UMaryland, UNebraska-Lincoln, Unesp_Brazil, UNSW_Australia, UPF_CRG_Barcelona, UPF_CRG_Barcelona, US_AFRL_CarrollHS, USMA-West_Point, USP-Brazil, UST_Beijing, USTC, UT-Knoxville, Utrecht, Valencia_UPV, Vilnius-Lithuania, Vilnius-Lithuania-OG, Virginia, Warwick, Washington, WashU_StLouis, Waterloo, Westminster_UK, WHU-China, William_and_Mary, Worldshaper-Wuhan, WPI_Worcester, XJTLU-CHINA, XJTU-China, XMU-China, Yale, ZJU-China, ZJUT-China (2020). Robust estimation of bacterial cell count from optical density. Communications Biology.

[R3] Bjerkan Geir, Witsø Eivind, Bergh Kåre (2009). Sonication is superior to scraping for retrieval of bacteria in biofilm on titanium and steel surfaces in vitro. Acta Orthopaedica.

[R4] Buckingham-Meyer Kelli, Miller Lindsey A., Parker Albert E., Walker Diane K., Sturman Paul, Novak Ian, Goeres Darla M. (2022). Harvesting and Disaggregation: An Overlooked Step in Biofilm Methods Research. Journal of Visualized Experiments.

[R5] Cámara Miguel, Green William, MacPhee Cait E., Rakowska Paulina D., Raval Rasmita, Richardson Mark C., Slater-Jefferies Joanne, Steventon Katerina, Webb Jeremy S. (2022). Economic significance of biofilms: a multidisciplinary and cross-sectoral challenge. npj Biofilms and Microbiomes.

[R6] Fedorowski A., Möller S.‐J., Melander O. (2013). Response to the letter by prof.
Dal Moro: the Dark Side of the Swoon– antihypertensive treatment in the elderly. Journal of Internal Medicine.

[R7] Fleming D, Chahin L, Rumbaugh K (2017). Glycoside Hydrolases Degrade Polymicrobial Bacterial Biofilms in Wounds.. Antimicrob Agents Chemother.

[R8] Fleming Derek, Redman Whitni, Welch Garrett S., Mdluli Nontokozo V., Rouchon Candace N., Frank Kristi L., Rumbaugh Kendra P. (2020). Utilizing glycoside hydrolases to improve the quantitation and visualization of biofilm bacteria. Biofilm.

[R9] Flemming Hans-Curt, van Hullebusch Eric D., Little Brenda J., Neu Thomas R., Nielsen Per H., Seviour Thomas, Stoodley Paul, Wingender Jost, Wuertz Stefan (2024). Microbial extracellular polymeric substances in the environment, technology and medicine. Nature Reviews Microbiology.

[R10] Folliero Veronica, Franci Gianluigi, Dell’Annunziata Federica, Giugliano Rosa, Foglia Francesco, Sperlongano Rossella, De Filippis Anna, Finamore Emiliana, Galdiero Massimiliano (2021). Evaluation of Antibiotic Resistance and Biofilm Production among Clinical Strain Isolated from Medical Devices. International Journal of Microbiology.

[R11] Fowler Teresa E., Bloomquist Ryan F., Sakhalkar Monali V., Bloomquist Doan Tam (2023). Chronic Purulent Conjunctivitis Associated With Extensively Drug-Resistant
*Pseudomonas aeruginosa*. JAMA Ophthalmology.

[R12] Goldufsky Josef, Wood Stephen J., Jayaraman Vijayakumar, Majdobeh Omar, Chen Lin, Qin Shanshan, Zhang Chunxiang, DiPietro Luisa A., Shafikhani Sasha H. (2015). *Pseudomonas aeruginosa*
uses T3SS to inhibit diabetic wound healing. Wound Repair and Regeneration.

[R13] Gonzalez Manuel R., Fleuchot Betty, Lauciello Leonardo, Jafari Paris, Applegate Lee Ann, Raffoul Wassim, Que Yok-Ai, Perron Karl (2016). Effect of Human Burn Wound Exudate on Pseudomonas aeruginosa Virulence. mSphere.

[R14] Hartmann Raimo, Jeckel Hannah, Jelli Eric, Singh Praveen K., Vaidya Sanika, Bayer Miriam, Rode Daniel K. H., Vidakovic Lucia, Díaz-Pascual Francisco, Fong Jiunn C. N., Dragoš Anna, Lamprecht Olga, Thöming Janne G., Netter Niklas, Häussler Susanne, Nadell Carey D., Sourjik Victor, Kovács Ákos T., Yildiz Fitnat H., Drescher Knut (2021). Quantitative image analysis of microbial communities with BiofilmQ. Nature Microbiology.

[R15] Holicheva Angelina A., Kozlov Konstantin S., Boiko Daniil A., Kamanin Maxim S., Provotorova Daria V., Kolomoets Nikita I., Ananikov Valentine P. (2025). Deep generative modeling of annotated bacterial biofilm images. npj Biofilms and Microbiomes.

[R16] Klinger-Strobel Mareike, Suesse Herbert, Fischer Dagmar, Pletz Mathias W., Makarewicz Oliwia (2016). A Novel Computerized Cell Count Algorithm for Biofilm Analysis. PLOS ONE.

[R17] Korshoj LE, Kielian T (2024). Bacterial single-cell RNA sequencing captures biofilm transcriptional heterogeneity and differential responses to immune pressure.. bioRxiv.

[R18] Kragh Kasper N., Hutchison Jaime B., Melaugh Gavin, Rodesney Chris, Roberts Aled E. L., Irie Yasuhiko, Jensen Peter Ø., Diggle Stephen P., Allen Rosalind J., Gordon Vernita, Bjarnsholt Thomas (2016). Role of Multicellular Aggregates in Biofilm Formation. mBio.

[R19] Martini K. Michael, Boddu Satya Spandana, Nemenman Ilya, Vega Nic M. (2024). Maximum likelihood estimators for colony-forming units. Microbiology Spectrum.

[R20] Mountcastle Sophie E., Vyas Nina, Villapun Victor M., Cox Sophie C., Jabbari Sara, Sammons Rachel L., Shelton Richard M., Walmsley A. Damien, Kuehne Sarah A. (2021). Biofilm viability checker: An open-source tool for automated biofilm viability analysis from confocal microscopy images. npj Biofilms and Microbiomes.

[R21] Redman WK, Welch GS, Williams AC, Damron AJ, Northcut WO, Rumbaugh KP (2021). Efficacy and safety of biofilm dispersal by glycoside hydrolases in wounds.. Biofilm.

[R22] Sauer Karin, Stoodley Paul, Goeres Darla M., Hall-Stoodley Luanne, Burmølle Mette, Stewart Philip S., Bjarnsholt Thomas (2022). The biofilm life cycle: expanding the conceptual model of biofilm formation. Nature Reviews Microbiology.

[R23] Schaber J. Andy, Triffo W. Jeffrey, Suh Sang Jin, Oliver Jeffrey W., Hastert Mary Catherine, Griswold John A., Auer Manfred, Hamood Abdul N., Rumbaugh Kendra P. (2007). *Pseudomonas aeruginosa*
Forms Biofilms in Acute Infection Independent of Cell-to-Cell Signaling. Infection and Immunity.

[R24] Stoodley P., Sauer K., Davies D. G., Costerton J. W. (2002). Biofilms as Complex Differentiated Communities. Annual Review of Microbiology.

[R25] Usui Masaru, Yoshii Yutaka, Thiriet-Rupert Stanislas, Ghigo Jean-Marc, Beloin Christophe (2023). Intermittent antibiotic treatment of bacterial biofilms favors the rapid evolution of resistance. Communications Biology.

[R26] Wang Jie, Tabassum Nazia, Toma Tanjin T, Wang Yibo, Gahlmann Andreas, Acton Scott T (2022). 3D GAN image synthesis and dataset quality assessment for bacterial biofilm. Bioinformatics.

[R27] Wang Shuai, Zhu Huiyan, Zheng Gexi, Dong Feng, Liu Chongxuan (2022). Dynamic Changes in Biofilm Structures under Dynamic Flow Conditions. Applied and Environmental Microbiology.

